# CULTIVATING AN ATTITUDE OF GRATITUDE: A BRIEF GRATITUDE INTERVENTION FOR OLDER ADULTS WITH CHRONIC PAIN

**DOI:** 10.1093/geroni/igad104.2579

**Published:** 2023-12-21

**Authors:** Shelley Condon, Brian Cox, Patricia Parmelee

**Affiliations:** The University of Alabama, Tuscaloosa, Alabama, United States; Alabama Research Institute on Aging, Tuscaloosa, Alabama, United States; The University of Alabama, Tuscaloosa, Alabama, United States

## Abstract

Gratitude interventions have emerged as a promising approach to ameliorate the negative impact of pain and enhance quality of life. Despite the high prevalence of older adults with chronic pain, there are very few gratitude intervention studies in this population. The current study examined the feasibility and effectiveness of a two-week daily gratitude journaling intervention in a sample of 38 older adults (M=67.53 years) with chronic knee or hip pain. Participants were randomly assigned to the gratitude group (n=21), who wrote down three things they were grateful for every day, or the attention-matched control group, who did not journal. All participants completed pre- and post-intervention interviews and received 14 nightly phone calls, allowing for “global” (pre/post interviews) and “daily” (nightly calls) analyses of the study aims. Aim 1 examined the effects of gratitude on well-being using regression and multilevel model analyses, while Aim 2 examined the effects of the intervention on changes in well-being using repeated measures analyses of variance and multilevel model analyses. In Aim 1, significant associations were found between trait and state gratitude and well-being, supporting previous research and a multifaceted conceptualization and measurement of gratitude. In Aim 2, significant main effects for time emerged, suggesting that participants’ well-being improved regardless of their treatment group. Income level was also a consistent predictor of well-being, demonstrating disparities in chronic pain. The impact of small sample size, COVID-19, and methodological limitations will be discussed. Future research plans and recommendations will also be provided.

